# Infant Behaviors, Prenatal Cocaine Exposure, and Adult Intelligence

**DOI:** 10.1001/jamanetworkopen.2024.11905

**Published:** 2024-05-17

**Authors:** Lynn T. Singer, Jeffrey M. Albert, Sonia Minnes, Meeyoung O. Min, June-Yung Kim

**Affiliations:** 1 Department of Population and Quantitative Health Sciences, School of Medicine, Case Western Reserve University, Cleveland, Ohio; 2 Jack, Joseph and Morton Mandel School of Applied Social Sciences, Case Western Reserve University, Cleveland, Ohio; 3 School of Social Work, University of Utah, Salt Lake City; 4 Department of Social Work College of Nursing and Professional Disciplines, University of North Dakota, Grand Forks

## Abstract

**Importance:**

Linking prenatal drug exposures to both infant behavior and adult cognitive outcomes may improve early interventions.

**Objective:**

To assess whether neonatal physical, neurobehavioral, and infant cognitive measures mediate the association between prenatal cocaine exposure (PCE) and adult perceptual reasoning IQ.

**Design, Setting, and Participants:**

This study used data from a longitudinal, prospective birth cohort study with follow-up from 1994 to 2018 until offspring were 21 years post partum. A total of 384 (196 PCE and 188 not exposed to cocaine [NCE]) infants and mothers were screened for cocaine or polydrug use. Structural equation modeling was performed from June to November 2023.

**Exposures:**

Prenatal exposures to cocaine, alcohol, marijuana, and tobacco assessed through urine and meconium analyses and maternal self-report.

**Main Outcomes and Measures:**

Head circumference, neurobehavioral assessment, Bayley Scales of Infant Development, Fagan Test of Infant Intelligence score, Wechsler Perceptual Reasoning IQ, Home Observation for Measurement of the Environment (HOME) score, and blood lead level.

**Results:**

Among the 384 mothers in the study, the mean (SD) age at delivery was 27.7 (5.3) years (range, 18-41 years), 375 of 383 received public assistance (97.9%) and 336 were unmarried (87.5%). Birth head circumference (standardized estimate for specific path association, −0.05, SE = 0.02; *P* = .02) and 1-year Bayley Mental Development Index (MDI) (standardized estimate for total of the specific path association, −0.05, SE = 0.02; *P* = .03) mediated the association of PCE with Wechsler Perceptual Reasoning IQ, controlling for HOME score and other substance exposures. Abnormal results on the neurobehavioral assessment were associated with birth head circumference (β = −0.20, SE = 0.08; *P* = .01). Bayley Psychomotor Index (β = 0.39, SE = 0.05; *P* < .001) and Fagan Test of Infant Intelligence score (β = 0.16, SE = 0.06; *P* = .01) at 6.5 months correlated with MDI at 12 months.

**Conclusions and Relevance:**

In this cohort study, a negative association of PCE with adult perceptual reasoning IQ was mediated by early physical and behavioral differences, after controlling for other drug and environmental factors. Development of infant behavioral assessments to identify sequelae of prenatal teratogens early in life may improve long-term outcomes and public health awareness.

## Key Points

**Question:**

Can infant behaviors mediate perceptual reasoning differences in adulthood associated with prenatal cocaine exposure?

**Findings:**

In this cohort study of 196 offspring prenatally exposed to cocaine and 188 nonexposed offspring followed up over 13 time points from birth to 21 years of age, birth head circumference and 1-year developmental outcome mediated the association between prenatal cocaine exposure and adult perceptual reasoning, after controlling for the home environment, prenatal use of other substances, and lead exposure.

**Meaning:**

This study suggests that assessment of early infant sensorimotor development and birth head circumference after prenatal drug exposure may provide a basis for early intervention to prevent deficits in adulthood.

## Teaser

This cohort study assesses whether neonatal physical, neurobehavioral, and infant cognitive measures mediate the association between prenatal cocaine exposure and adult perceptual reasoning.

## Introduction

Understanding the association of prenatal drug exposures with adult outcomes is difficult due to barriers in demonstrating causal relationships between exposures and adult behavior. Among these barriers are the lack of cohort studies after adolescence due to attrition of families at high risk and difficulty in maintaining longitudinal research funding.^1^ Developmental outcomes are influenced by environmental factors that may ameliorate or exacerbate early deficits,^2^ making drug effects less clear. Neonatal assessments closest to fetal exposure are procedurally difficult given the lack of stability in infant behaviors and poor predictive validity of preverbal infant tests.^3,4^ Promising neonatal assessments have now been identified that capture early physiological and neurobehavioral markers associated with later behavior.^5^ Identifying markers of prenatal drug exposures may lead to efficient studies that provide timely public health notification about teratogens and interventions earlier than school age.

This analysis used data from a large prospective cohort study of prenatal cocaine exposure (PCE) following up infants from birth at 13 time points through 21 years of age.^6,7^ Infants exposed prenatally to cocaine or other drugs were identified through maternal self-report, urine biomarkers, or meconium biomarkers. A comparison cohort of similar race and ethnicity, socioeconomic status, and risk status who were not exposed to cocaine (NCE) was formed. Infants were assessed neonatally and followed up with cognitive, motor, growth, social-emotional, and behavioral assessments, with exceptional retention from first to last follow-up at age 21 years (96%-82%).^7^ Confounding factors included other substance exposures,^6,7,8^ caregiving environment,^9^ maternal psychological distress,^6,7,8,9,10^ and lead exposure.^11,12^

## Methods

### Sample and Procedures

This study included 384 infants (196 PCE and 188 NCE) and their mothers followed up from birth (1994) to 21 years of age (2018) from a Cleveland, Ohio, county hospital,^6,7^ identified from a population screened with urine or meconium for benzoylecgonine, meta-hydroxy benzoylecgonine, cannabinoids, opiates, phencyclidine, amphetamines, cocaethylene, and benzodiazepines. Exclusions included women with a psychiatric history, low intellectual functioning, HIV positivity, or chronic medical illness, as well as infants with Down syndrome, fetal alcohol syndrome, or congenital heart defects. A nurse recruiter approached 647 women, with 54 excluded. One hundred fifty-five mothers refused to participate (49 PCE and 106 NCE), and 23 mothers (9 PCE and 14 NCE) did not come to the enrollment visit. Mothers who refused to participate were more likely than participants to be non–cocaine users and younger; race and ethnicity, sex, and infant birth characteristics did not differ. Of 415 enrolled, 218 were PCE (any positive screening result [urine, meconium, and self-report]). Infants with negative screening results for all were NCE but were exposed to other substances (alcohol, tobacco, and marijuana). After enrollment, 14 children (10 PCE and 4 NCE) died. At 21 years of age, data were available for 325 of 401 living participants (81.0%). The Case Western Reserve University institutional review board approved all procedures and visits. Participants received a monetary stipend, lunch, and transportation. A certificate of confidentiality (DA-09-146) was obtained. Written informed consent was obtained. For children, consent was obtained from the caregiver, and assent was obtained from the child starting at 7 years of age. Children and caregivers were assessed by separate examiners masked to exposure. The current caregiver was interviewed if the birth mother was not the caregiver. For all child, adolescent, or adult outcomes, assessors were master’s level or equivalent psychology assistants supervised by licensed psychologists. For children younger than 2 years, tests were administered at ages corrected for preterm birth. This report followed the Strengthening the Reporting of Observational Studies in Epidemiology (STROBE) reporting guideline.

### Primary Cognitive Outcome

Our primary focus was perceptual reasoning, a nonverbal cognitive executive function reflecting abstract reasoning,^13^ negatively associated with PCE in our cohort from 4 to 21 years of age^7,9,14,15,16,17,18^ and in others.^19,20^ We used structural equation modeling to assess whether the association of perceptual reasoning IQ (PRIQ) with PCE was mediated through infant behaviors negatively associated with PCE, controlling for confounders.^6,21,22,23,24^

### Infant Outcomes

Neonatal or infant physiological and behavioral measures included birth head circumference (HC) in centimeters, extracted from hospital records^6^; occurrence of neonatal neurobehavioral abnormalities^21^; neonatal visual preferences^22^; visual recognition memory at 6.5 and 12 months; motor development at 6.5 months; and mental development at 12 and 24 months.^24,25^ Covariates included level of prenatal exposure to tobacco, marijuana, and alcohol; quality of the caregiving environment at 4 years of age^14,26^; and preschool lead exposure.^11^

### Maternal Measures

#### Prenatal Cocaine and Other Substance Exposures

A research assistant interviewed mothers about prenatal drug use with a timeline follow-back method, details of which can be found in a prior publication.^6^ This method yields summary measures of frequency, timing, and amount of drug use by trimester and over the pregnancy, which were combined with meconium screening results to determine classification dichotomously (PCE or NCE) or intensity of cocaine exposure (heavier or lighter).

#### Other Maternal or Caregiver Measures

Maternal race and ethnicity (using National Institutes of Health classification), educational level, and socioeconomic status (public assistance vs none), covariates known to be associated with cognitive outcomes, were determined by self-report. The Global Severity Index, a summary scale of the Brief Symptom Inventory,^27^ measured psychological distress.

### Infant or Child Measures

#### Neurobehavioral Assessment

The Neurobehavioral (NB) Assessment,^21,28^ administered between 37 and 50 weeks of age, assesses visual or auditory orienting, passive tone and reflexes, head control, movement quality, and tone of extremity movements. It has demonstrated validity to identify risk from abnormal brain-behavior associations and has excellent reliability. The examination rates behavior categories as normal (0) or abnormal (1). For analyses, infants were characterized as having no abnormalities or any category abnormality.^21,28^

#### Neonatal Visual Preferences

Neonatal visual preferences, a measure of visual perception and attention, were tested using an experimental paired comparison task.^22,29,30,31,32^ The testing apparatus consisted of a box structure with a pivoting stage.^33^ The infant and mother sat on one side of the apparatus with the observer on the opposite side. The stage was opened by the observer, and stimuli were attached outside of the infant’s view. A peephole halfway between the 2 stimuli allowed the observer to record the infant’s visual fixations, with a digital timer measuring fixation length. The assessment consisted of 4 pairs of visual stimuli, illustrated by Singer et al.^22^ The assessment yields a percentage novelty score (range, 0-100; scores ≤50 indicate chance performance and higher scores indicate greater attention), the ratio of time spent looking at the novel stimulus vs total looking time for each problem. The score for each of the first 2 problems was used to compute a mean preference score for each infant.

#### The Fagan Test of Infant Intelligence

At 6.5 and 12 months, the Fagan Test of Infant Intelligence (FTII) was administered.^23^ The FTII is a standardized test of intelligence^34,35^ using the paired comparison procedure described in a series of 10 developmentally appropriate tasks whereby 1 picture is presented to the infant during a familiarization period and presented again alongside a novel picture for a trial period after the infant has accumulated a predetermined amount of time looking at the familiarization stimulus. Pictures were black and white or color photographs of faces of men, women, and infants.^33,34^ A novelty score is obtained by dividing the amount of time (in seconds) that the infant looks at the novel picture by the total time looking at both pictures during the trial. A mean percentage novelty score (range, 0-100; scores ≤50 indicate chance performance and higher scores indicate higher visual recognition memory and intelligence) is calculated by averaging the 10 items. Tests of visual recognition memory demonstrate good predictive and discriminant validity^3^ and have greater predictive validity for later cognitive performance than standard infant sensorimotor assessments.^3,36^

#### The Bayley Scales of Infant Development

At 6.5, 12, and 24 months, infants were administered the Bayley Scales of Infant Development.^25^ The scales yield a Mental Development Index (MDI), a standard score reflecting memory, language, and problem-solving abilities, and a Psychomotor Development Index (PDI) of gross and fine motor control and coordination. Normative data yield a mean (SD) score of 100 (15).

#### Perceptual Reasoning Assessments

At 4 years, children were administered the Wechsler Preschool and Primary Scales of Intelligence–Revised,^14,16^ a standardized, normative measure of intelligence that yields full scale, verbal, and performance IQs. The performance scale was used for the present analysis. At 9 and 15 years,^9,15^ we administered the Wechsler Intelligence Scale for Children–Fourth Edition (WISC IV),^17^ yielding a summary PRIQ with substantial reliability and validity. At 21 years,^7^ the Wechsler Abbreviated Scale of Intelligence–Second Edition^18^ provided a brief, reliable estimate of general cognitive ability, including perceptual reasoning. The summary PRIQ was used at 9, 15, and 21 years of age.

#### Caregiver Quality and Lead Exposure

Quality of the caregiving environment was assessed through an interview at 4 years with the Home Observation for Measurement of the Environment (HOME) Preschool version.^26^ Blood lead levels were obtained at 2 and 4 years of age.^11^ The numbers of children with valid measures at 2 and 4 years were 143 and 274, respectively, with mean values used for children seen at both ages.

### Statistical Analysis

Structural equation modeling was performed from June to November 2023. Log transformation normalized skewed data (drug exposures, maternal psychological distress, and blood lead levels). Univariate descriptive statistics were based on the original distribution; bivariate or multivariate analyses used normalized data. Sample characteristics were compared by PCE using the *t* test and the χ^2^ test. We examined bivariate associations using zero-order Pearson correlations.

Mediation effects of PCE were examined by fitting a structural equation model. An extended linear growth curve model (combined with a path model) was fit (Figure), representing hypothesized mechanisms of the associations of PCE, while excluding superfluous variables. The growth curve function involved a latent intercept and slope, measured by the repeated measures (at 4, 7, 15, and 21 years of age) of PRIQ. Potential mediators were modeled via linear regression except for “any abnormality,” modeled via probit regression. The structural equation model allowed for a correlation between PDI and FTII score at 6.5 months, as these variables are contemporaneous. Estimation of model coefficients and inference was conducted using weighted least squares with a diagonal weight matrix, with SEs and mean values and variance-adjusted χ^2^ test statistics using a full weight matrix, along with the theta parameterization in MPlus, version 8.3 (Muthén & Muthén)^37^ for the categorical variable (NB). Standardized coefficients were tested using 2-sided *z* score tests. Aside from PCE, the main variables of interest, prenatal tobacco, alcohol, and marijuana exposure, were included in the model for each outcome (including intermediate variables). The HOME score was included as a covariable for intercept and slope.

**Figure.**
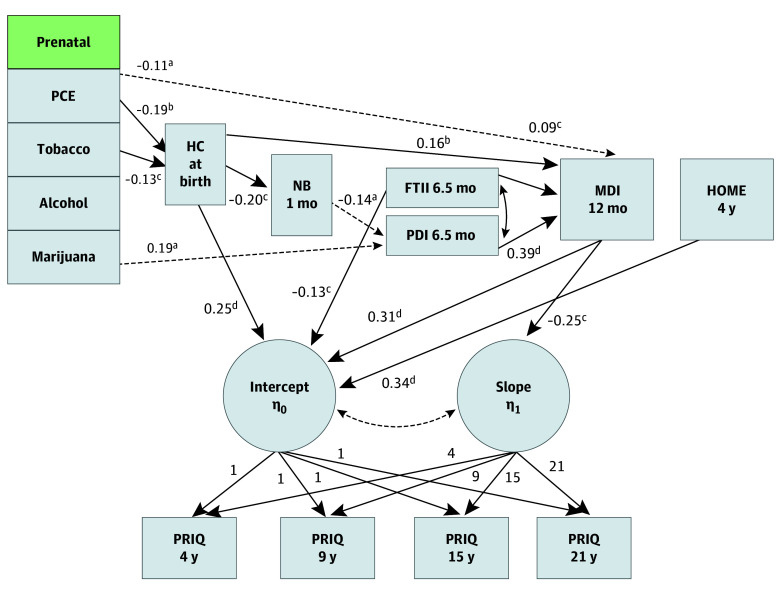
Structural Model of Birth Head Circumference (HC) and Infant Neurodevelopment as Mediators of the Association of Prenatal Cocaine Exposure (PCE) to Adult Perceptual Reasoning Latent growth curve modeling on the associations of PCE with children’s perceptual reasoning IQ (PRIQ) over time from childhood to emerging adulthood. Rectangles indicate observed variables, and circles indicate latent constructs. Values indicate standardized path coefficients except for intercept and slope coefficients, which are the fixed scores. Single-arrow lines indicate path coefficients, whereas double-arrow lines indicate correlations. Solid lines indicate statistically significant coefficients (*P* < .05), whereas dotted lines indicate marginally significant coefficients (.05 < *P* < .10). Lines for nonsignificant coefficients are removed (*P* > .10); a line indicating a correlation between the intercept and slope was retained, although nonsignificant (*P* = .14). FTII 6.5 mo indicates Fagan Test of Infant Intelligence score at 6.5 months; HOME 4 y, Home Observation for Measurement of the Environment score at 4 years; MDI 6.5 mo, Mental Development Index at 6.5 months; NB 1 mo, Neurobehavioral Assessment at 1 month; PDI 6.5 mo, Psychomotor Development Index at 6.5 months; and PRIQ, Perceptual Reasoning IQ. ^a^*P* < .10. ^b^*P* < .01. ^c^*P* < .05. ^d^*P* < .001.

Alternative versions were fit with lead exposure as a covariate, using the χ^2^ goodness-of-fit test, the Comparative Fit Index, the Tucker-Lewis Index, the root mean square error of approximation, and the standardized root mean square residual.^38^ Indirect associations were estimated as products of coefficients and tested using *z* score tests based on delta method estimates of SE. Corresponding 95% CIs were computed. Analysis assumed missing data were missing at random. All *P* values were from 2-sided tests, and results were deemed statistically significant at *P* < .05. All analyses were conducted using MPlus, version 8.3 (Base Program and Combination Add-On) (Muthén & Muthén).^37^

## Results

### Descriptive Statistics

Among the 384 mothers in the study, the mean (SD) age at delivery was 27.7 (5.3) years (range, 18-41 years); 0.5% were Asian (2 of 384), 79.9% were Black (307 of 384), 3.4% were Hispanic (13 of 384), 12.8% were White (49 of 384), and 6.8% were biracial or multiracial (26 of 384) (maternal race and ethnicity were calculated based child data in Table 1); 375 of 383 were receiving public assistance (97.9%); and 336 were unmarried (87.5%). A total of 154 mothers (40.1%) had not finished high school (mean [SD] years of schooling, 11.8 [1.6] years; range, 7-19 years).

**Table 1.** Child and Mother or Caregiver Characteristics by Cocaine Status (N = 384)

Characteristic	PCE (n = 196)	NCE (n = 188)	*P* value
**Child**			
Sex, No. (%)			
Male	89 (45.4)	93 (49.5)	.43
Female	107 (54.6)	95 (50.5)	
Race, No. (%)			
Asian	1 (0.5)	1 (0.5)	.87
Black	160 (81.6)	147 (78.2)	
White	23 (11.7)	26 (13.8)	
≥1 Race	12 (6.1)	14 (7.4)	
Ethnicity, No. (%)			
Hispanic	8 (4.1)	5 (2.7)	.44
Non-Hispanic	188 (95.9)	183 (97.3)	
Head circumference, mean (SD), cm	32.3 (2.1)	33.5 (2.4)	<.001
Cocaine in meconium, mean (SD), ng	150.7 (441.9)	NA	NA
Cocaethylene in meconium, mean (SD), ng	17.7 (62.7)	NA	NA
Benzoylecgonine in meconium, mean (SD), ng	596.6 (1543.4)	NA	NA
Lead level at 2 and 4 y, mean (SD), µg/dL	7.0 (4.1)	8.0 (4.6)	.04
Neonatal visual preference score, mean (SD)	56.7 (15.2)	58.0 (14.8)	.58
NB abnormal, No./total No. (%)	51/141 (36.8)	46/154 (29.9)	.27
FTII score at 6.5 mo, mean (SD)	57.7 (8.0)	58.7 (8.3)	.32
FTII score at 1 y, mean (SD)	56.8 (7.6)	59.0 (6.4)	.008
PDI score at 6 mo, mean (SD)	94.9 (14.1)	96.8 (13.3)	.61
MDI score at 1 y, mean (SD)	95.0 (10.0)	99.3 (10.4)	<.001
MDI score at 2 y, mean (SD)	83.1 (13.3)	89.1 (13.8)	<.001
WPPSI-R Performance IQ at 4 y, mean (SD)	84.8 (14.6)	88.0 (15.1)	.03
WISC-IV Perceptual Reasoning IQ at 9 y, mean (SD)	87.2 (13.6)	91.2 (14.1)	.005
WISC-IV Perceptual Reasoning IQ at 15 y, mean (SD)	86.4 (13.5)	90.1 (13.8)	.01
WASI-II Perceptual Reasoning IQ at 21 y, mean (SD)	87.5 (11.5)	91.4 (14.1)	.01
**Mother or caregiver**			
Prenatal substance use			
Cigarettes/d, mean (SD)	11.6 (11.1)	4.1 (7.7)	<.001
Alcohol drinks/wk, mean (SD)^a^	9.6 (17.3)	1.4 (4.5)	<.001
Marijuana joints/wk, mean (SD)^b^	0.6 (1.3)	0.2 (0.9)	<.001
Cocaine units/wk, mean (SD)^c^	24.0 (45.5)	NA	NA
HOME score at 4 y, mean (SD)	42.0 (6.4)	41.7 (6.7)	.64

Abbreviations: FTII, Fagan Test of Infant Intelligence; HOME, Home Observation for Measurement of the Environment; MDI, Mental Development Index; NA, not applicable; NB, Neurobehavioral Assessment; NCE, no prenatal cocaine exposure; PCE, prenatal cocaine exposure; PDI, Psychomotor Development Index; WASI-II, Wechsler Abbreviated Scale of Intelligence–2nd Edition; WISC-IV, Wechsler Intelligence Scale for Children–4th Edition; WPPSI-R, Wechsler Preschool and Primary Scale of Intelligence–Revised.

^a^ Mean drinks of beer, wine, or hard liquor per week, each equivalent to 0.5 mL of absolute alcohol.

^b^ Mean number of joints of marijuana per week.

^c^ Mean cocaine units per week.

A total of 196 children were prenatally exposed to cocaine, 278 (72.4%) were prenatally exposed to tobacco, 248 (64.6%) were prenatally exposed to alcohol, and 104 (27.1%) were prenatally exposed to marijuana. Infants’ mean (SD) birth weight was 2904.7 (695.6) g, and the mean (SD) HC was 32.9 (2.3) cm. Compared with children with NCE, children with PCE had smaller birth HC and were prenatally exposed to more tobacco, alcohol, and marijuana (Table 1). The percentage of infants with any abnormality on the NB Assessment did not differ by group, nor did visual preference scores. However, when groups were categorized into heavier vs lighter exposure to cocaine, there were reliable differences in visual preferences (mean [SD] score: heavy exposure, 54.1 [15.7] vs light exposure, 61.0 [13.6] vs no exposure, 57.9 [14.8]; *P* < .04) and in the percentage of infants with any abnormality (heavy exposure, 39 of 82 [47.6%] vs light exposure, 21 of 76 [27.6%] vs no exposure, 49 of 161 [30.4%]; *P* < .02), as per prior publications.^21,22^ Children with PCE had lower MDI and PDI scores than those with NCE. Children with NCE had higher lead levels than children with PCE, while HOME scores did not differ between groups. Children with PCE had lower PRIQ scores at 4, 9, 15, and 21 years of age than children with NCE.

### Correlations of Prenatal Drug Measures, Outcome Measures, and Covariates

Table 2 presents the associations of prenatal, birth, and infancy measures with each other, with covariates, and with the PRIQ at all ages. Neonatal visual preferences and 12-month FTII score were associated with PCE but not associated with any other measure and were removed from the model. The FTII score at 6.5 months remained in the model.

**Table 2.** Correlations Among Key Observed Variables

Variable	Prenatal tobacco use^a^	Prenatal alcohol use^a^	Prenatal marijuana use^a^	HC, cm	Lead level, µg/dL	HOME score at 4 y	Neonatal visual preference score	NB abnormal	FTII score at 6.5 mo	FTII score at 1 y	PDI score at 6 mo	MDI score at 1 y	MDI score at 2 y	PRIQ at 4 y	PRIQ at 9 y	PRIQ at 15 y	PRIQ at 21 y
PCE	0.49^b^	0.49^b^	0.22^b^	−0.26^b^	−0.12^c^	0.02	−0.04	0.06	−0.06	−0.15^c^	−0.07	−0.20^b^	−0.22^b^	−0.11^d^	−0.15^c^	−0.14^c^	−0.15^c^
Prenatal tobacco use^a^		0.43^b^	0.21^b^	−0.20^b^	0.02	0.01	−0.02	0.06	−0.02	−0.07	−0.09^e^	−0.11^e^	−0.12^d^	−0.06	−0.04	−0.05	−0.04
Prenatal alcohol use^a^			0.14^c^	−0.11^d^	0.03	−0.01	−0.02	0.00	−0.10^e^	−0.01	0.01	−0.11^d^	−0.18^c^	−0.08	−0.10^e^	−0.10^e^	−0.12^d^
Prenatal marijuana use^a^				−0.10^d^	−0.05	0.04	−0.03	−0.03	0.01	0.00	0.07	−0.05	−0.04	0.00	0.05	−0.00	−0.05
HC, cm					−0.10^e^	−0.08	0.08	−0.19^c^	0.09	0.09	0.07	0.20^d^	0.19^b^	0.21^b^	0.25^b^	0.21^b^	0.24^b^
Lead level, µg/dL						−0.30^b^	−0.02	−0.04	−0.01	0.03	0.01	0.08	−0.13^d^	−0.31^b^	−0.17^c^	−0.19^c^	−0.25^b^
HOME score at 4 y							0.02	−0.04	0.05	0.04	0.05	0.06	0.23^b^	0.32^b^	0.19^b^	0.24^b^	0.23^b^
Neonatal visual preference score								−0.10	−0.11	−0.10	0.04	−0.01	0.02	0.03	0.00	0.04	0.05
NB abnormal									0.02	−0.05	−0.13^d^	−0.13^d^	−0.12^e^	−0.04	−0.05	−0.07	0.03
FTII score at 6.5 mo										0.17^d^	−0.07	0.17^c^	0.00	0.02	−0.03	−0.01	0.04
FTII score at 1 y											0.05	0.06	0.00	0.04	0.01	−0.08	0.00
PDI score at 6 mo												0.40^b^	0.26^b^	0.12^d^	0.15^d^	0.15^c^	0.09
MDI score at 1 y													0.47^b^	0.28^b^	0.24^b^	0.24^b^	0.16^c^
MDI score at 2 y														0.50^b^	0.33^b^	0.34^b^	0.28^b^
PRIQ at 4 y															0.58^b^	0.55^b^	0.60^b^
PRIQ at 9 y																0.73^b^	0.70^b^
PRIQ at 15 y																	0.79^b^
PRIQ at 21 y																	

Abbreviations: FTII, Fagan Test of Infant Intelligence; HC, head circumference; HOME, Home Observation for Measurement of the Environment; MDI, Mental Development Index; NB, Neurobehavioral Assessment; PCE, prenatal cocaine exposure; PDI, Psychomotor Development Index; PRIQ, Perceptual Reasoning IQ.

^a^ Log transformed.

^b^ *P* < .001.

^c^ *P* < .01.

^d^ *P* < .05.

^e^ *P* < .10.

Lead exposure^9,11,12^ was associated with PRIQ, but exposure was lower in the PCE group and could not mediate the association of PCE with PRIQ. The HOME score^9,14^ was associated with PRIQ but not associated with PCE, and thus it was not considered a mediator. However, we retained the HOME score in the model to ensure that a control measure reflecting socioeconomic status, parental educational level, and caregiving quality was included. Summary measures of prenatal alcohol, marijuana, and tobacco exposure were retained.

### Model Fit or Estimation

The model provided a good fit (Figure) (with an analysis sample size of 360): χ^2^_30_ = 39.78; *P* = .11; a Comparative Fit Index of 0.98; a Tucker-Lewis Index of 0.96; a root mean square error of approximation of 0.03 (90% CI, 0.00-0.05); and a standardized root mean square residual of 0.16. The Figure indicates the least marginally significant path coefficients. The fit model is the saturated version of direct association links of PCE in the model (Figure), with the exception of the HOME score, specified to be associated with the latent scores for PRIQ only. A correlation between the latent intercept and slope for PRIQ was not significant (*r* = −0.23; *P* = .14).

Prenatal cocaine exposure was associated with lower birth HC (β = −0.19, SE = 0.07; *P* = .01) and MDI (β = −0.11, SE = 0.06; *P* = .05) at 12 months (Figure). Birth HC was associated with the occurrence of abnormalities neonatally on the NB assessment (β = −0.20, SE = 0.08; *P* = .01) and directly with the MDI at 12 months (β = 0.09, SE = 0.04; *P* = .01) and with the overall PRIQ (β = 0.25, SE = 0.05; *P* < .001). The PDI at 6.5 months was associated with the MDI at 12 months (β = 0.39, SE = 0.05; *P* < .001), while the MDI at 12 months was associated both with PRIQ (β = 0.31, SE = 0.07; *P* < .001) and with the slope of PRIQ (β = −0.25, SE = 0.12; *P* = .03). An estimated 0.87-cm decrease in the mean HC and an estimated 2.2 decrease in the 12-month mean MDI score were found for children with PCE vs those with NCE.

In addition, other drug, socioeconomic, and environmental factors added to PRIQ outcome. Prenatal tobacco exposure was associated with smaller birth HC (β = −0.13, SE = 0.07; *P* = .04), and the HOME score was associated with higher PRIQ (β = 0.34, SE = 0.06; *P* < .001). A 1-unit increase in standardized prenatal tobacco exposure was associated with a 0.13 decrease in the mean standardized PRIQ, and a 1-unit decrease in the standardized HOME score was associated with a 0.34 increase in the mean standardized PRIQ. The Fagan Test of Infant Intelligence score at 6.5 months was associated with the MDI at 12 months (β = 0.16, SE = 0.06; *P* = .01) and PRIQ (β = −0.13, SE = 0.07; *P* = .05). A 1-unit increase in standardized lead level was associated with an 0.28 decrease in mean PRIQ (*P* < .001).

There were 2 reliable mediation effects. Birth HC was a mediator of the association of PCE with PRIQ (standardized estimate for specific path association, −0.05, SE = 0.02; *P* = .02). The MDI at 12 months was also a mediator of the association of PCE with PRIQ (standardized estimate for total of the path-specific associations, −0.05, SE = 0.02; *P* = .03). The total indirect association involves multiple specific paths through the MDI at 12 months, none of which reached significance. These mediation effects are in the expected direction because PCE is negatively associated with birth HC and MDI at 12 months, each of which is positively associated with overall PRIQ, such that birth HC and MDI at 12 months mediate the negative association of PCE with PRIQ.

## Discussion

Negative sequelae of PCE on perceptual reasoning were linked to poorer early physical growth and sensorimotor skills independent of other drug and environmental factors, suggesting a biological association of PCE with adult outcomes not previously demonstrated in teratology studies of prenatal exposures. Through physical and behavioral assessments to 21 years of age, we found that birth HC and 1-year neurodevelopmental outcome mediate the association of prenatal drug exposures (cocaine and tobacco) with lower scores in perceptual reasoning. Even earlier markers of abnormalities on a neurobehavioral assessment at 1 month and poorer performance in motor development and visual recognition memory at 6.5 months were associated with birth HC and 1-year outcome.

Abnormalities on neonatal assessment included attention, motor, tone, sensory differences, and jitteriness, while the 6.5-month PDI assessment indicated poorer fine and gross motor performance. The visual recognition memory test assesses visual-perceptual abstraction similar to the perceptual reasoning tasks at 4 years. Findings were surprising, as standard infancy tests of sensorimotor skills prior to 18 months of age have not been associated with later development,^3,4,39^ although recent data suggest this view is being reconsidered.^5,40^

### Strengths and Limitations

Strengths of the study include the prospective cohort design, the use of biomarkers and interviews for classification, high retention, control for other prenatal substance exposures and home environment, large sample size, and use of valid, standardized, reliable assessments across multiple ages. This study also has some limitations. The low socioeconomic status of the sample may limit generalizability of the findings. Moreover, other environmental exposures, in addition to lead, that may be associated with outcome were not measured. We also did not control for substance use at older ages that might have affected cognitive functions. However, a prior study of this cohort at 15.5 years found that adolescent substance use was associated with attention and impulsivity but not with PRIQ, mitigating that concern.^15^

## Conclusions

Our findings suggest that early neurobehaviors are markers of poorer adult perceptual reasoning after prenatal drug exposure, which was stable by 4 years of age, while the strong association of the home environment underscores potential for malleability. Combining physical and behavioral markers can potentially produce algorithms to identify infants in need of intervention, a continuing challenge in pediatric care.^4^ The associations of lead exposure and the HOME score with outcomes illustrate the significant additional association of social determinants of health with cognition. Further development of early infant neurobehavioral and cognitive tests, such as the NICU Network Neurobehavioral Scale^5^ and the FTII,^34^ to increase reliability and validity, with linkage to neuroimaging of brain development and long-term cognitive outcomes, may provide a basis for early interventions in at-risk populations and provide timely public health warnings about teratogens.

## Notes

Supplement.
Data Sharing Statement
