# Supplementary material for: Infant Behaviors, Prenatal Cocaine Exposure, and Adult Intelligence
Source: JAMA Netw Open. 2024 May 17;7(5):e2411905. doi: 10.1001/jamanetworkopen.2024.11905 (PMC11102018; doi:10.1001/jamanetworkopen.2024.11905)
Supplement: Supplement. — Data Sharing Statement [file jamanetwopen-e2411905-s001.pdf]

Data Sharing Statement

Singer. Infant Behaviors, Prenatal Cocaine Exposure, and Adult Intelligence. *JAMA Netw Open*. Published May 17, 2024. doi:10.1001/jamanetworkopen.2024.11905

Data
Data available: Yes
Data types: Deidentified participant data, Data dictionary
How to access data: lynn.singer@case.edu
When available: With publication

Supporting Documents
Document types: None

Additional Information
Who can access the data: researchers whose proposed use of the data has been approved
Types of analyses: Data will be available for a specified research purpose after approval of a proposal with a signed data access agreement.
Mechanisms of data availability: after approval of a proposal with a signed data access agreement.
Any additional restrictions: none
